# Starch-g-Acrylic Acid/Magnetic Nanochitin Self-Healing Ferrogels as Flexible Soft Strain Sensors

**DOI:** 10.3390/s23031138

**Published:** 2023-01-19

**Authors:** Pejman Heidarian, Abbas Z. Kouzani

**Affiliations:** School of Engineering, Deakin University, Geelong 3216, Australia

**Keywords:** magneto-responsive ferrogel, self-healing properties, magnetic nanochitin, soft strain sensor

## Abstract

Mechanically robust ferrogels with high self-healing ability might change the design of soft materials used in strain sensing. Herein, a robust, stretchable, magneto-responsive, notch insensitive, ionic conductive nanochitin ferrogel was fabricated with both autonomous self-healing and needed resilience for strain sensing application without the need for additional irreversible static chemical crosslinks. For this purpose, ferric (III) chloride hexahydrate and ferrous (II) chloride as the iron source were initially co-precipitated to create magnetic nanochitin and the co-precipitation was confirmed by FTIR and microscopic images. After that, the ferrogels were fabricated by graft copolymerisation of acrylic acid-g-starch with a monomer/starch weight ratio of 1.5. Ammonium persulfate and magnetic nanochitin were employed as the initiator and crosslinking/nano-reinforcing agents, respectively. The ensuing magnetic nanochitin ferrogel provided not only the ability to measure strain in real-time under external magnetic actuation but also the ability to heal itself without any external stimulus. The ferrogel may also be used as a stylus for a touch-screen device. Based on our findings, our research has promising implications for the rational design of multifunctional hydrogels, which might be used in applications such as flexible and soft strain sensors, health monitoring, and soft robotics.

## 1. Introduction

As science has progressed, wearable devices have gained popularity for use in fields including human movement detection, healthcare monitoring, and soft robotics. However, equipment designed to accommodate the growing number of smart wearable devices presents a difficulty when it comes to matching mechanical deformations such as bending, folding, twisting, and stretching [1,2,3,4,5]. Thanks to their flexibility and low weight, soft strain sensors have emerged as a leading candidate for future wearable devices. In comparison, traditional devices made from metals or semiconductors have a strain sensing range of less than 5% and are so stiff that they are uncomfortable to wear [4]. On the other hand, the current available soft, flexible strain sensors tend to fail unexpectedly and irreversibly due to mechanical stress and damage, thus losing their functionality [6,7].

Certain natural systems, however, have evolved means to restore themselves when they fail. The human skin is a prime example of a self-repairing natural system that can gain its initial properties after damage [4,8]. Inspired by this unique feature, in this research, we designed and developed a self-healing hydrogel constructed of magnetic nanochitin that can self-heal faster than human skin and might be used as a soft, flexible, self-healing strain sensor. As soft, wet, 3D crosslinked, hydrophilic polymers with unique properties, hydrogels have demonstrated their versatility in many research and industry fields, including medical applications [9,10], sensors [8,11,12], and water treatment [13]. However, the delicate and brittle nature of hydrogels makes their potential applications in wearable devices problematic. Thus far, many attempts have been made to solve these difficulties by mechanically reinforcing hydrogels using methods such as double networks and interpenetrating networks. Hydrogel toughening research in the last several years has shown that a number of different types of hydrogels, including nanocomposite hydrogels, double crosslinked hydrogels, and double network hydrogels, all have robust mechanical properties and can withstand significant deformation [14,15,16]. However, they ultimately break at a specific strain and irreversibly damage before any apparent fractures appear because of the presence of irreversible static chemical crosslinks inside their 3D networks [17,18,19]. Hydrogels, on the other hand, may have their networks prepared to self-repair in the case of break or damage by using reversible, dynamic physical or chemical crosslinks within their 3D structures. Hence, hydrogels fabricated by reversible, dynamic physical or chemical cross-linkers seem to be promising soft materials for flexible soft strain sensing due to their capacity to self-heal, especially when this fascinating feature is combined with ductility and conductivity [19,20,21].

However, owing to the inherent unstable mobile connections of dynamic crosslinks, ductility and toughness may drastically drop while fabricating self-healing hydrogels for soft, flexible wearable strain sensors, and the fabricated hydrogels may demonstrate a compromise between static and dynamic crosslinks in terms of mechanical strength and self-healing properties. Therefore, it is necessary to use additional chemical crosslinks in order to create a conductive hydrogel with repeatable stretchability, high toughness, and autonomous self-healing ability for strain-sensing applications [22,23]. In this work, a magnetic-field-sensitive hydrogel containing iron oxide (Fe_3_O_4_) nanoparticles, called ferrogel, is synthesised with a novel magnetic remote control strain sensing ability using nanochitin as a template through in situ hydrolysis of metal precursors, imparting both autonomous self-healing and toughness to a graft copolymerised acrylic acid/starch-based ferrogel without the need for additional chemical crosslinks. Fe_3_O_4_ nanoparticles have recently piqued the scientific community’s interest as biosensors [24], biolabelling [25], and targeted drug delivery [26] due to their enormous surface area, nano-size, excellent biocompatibility, low toxicity, and excellent physicochemical stability. However, agglomeration happens when Fe_3_O_4_ nanoparticles are utilised in polymers due to their large surface area and high interaction energy. To address this issue, templates for the manufacture of Fe_3_O_4_ nanoparticles may be used [27]. Because of its highly crystallised structure, great mechanical strength, large surface area, high aspect ratio, and abundance of functional groups accessible for modification, nanochitin, the second most abundant biopolymer on the planet, appears to be an ideal template on which Fe_3_O_4_ nanoparticles can be synthesised and grafted [28].

Nanochitin is a primary precursor of cationic polysaccharides found in nature and displays biocompatibility, biodegradability, minimal allergenicity, high mechanical strength, and colloidal properties in dispersed media. It is a long-chain linear polymer with repeating β(1,4)-*N*-acetylglucosamine units, a derivative of glucose [29]. However, nanochitin, despite its great potential, has remained yet an underutilised biomass resource compared to nanocellulose, the most abundant biopolymer in nature. This research, hence, can pave the way for its use in performance improvement and provide ideas as a building block for the creation of new materials and alternative templates onto which other nanoparticles, such as Fe_3_O_4_, can be synthesised. In order to do so, magnetic nanochitin, synthesised from shrimp shell, was incorporated into a graft-copolymerised acrylic acid-g-starch-based network as both nanofiber reinforcement and physical cross-linker to create a tough, elastic, magneto-responsive, self-healing, ionic conductive ferrogel with excellent strain sensing properties. This method endows our ferrogel with not only exceptional self-healing properties but also the mechanical strength necessary for strain-sensing applications, in addition to ionic conductivity and magneto-responsive properties.

## 2. Materials and Methods

### 2.1. Chemicals and Materials

Nanochitin, isolated from shrimp shells, was generously supplied by the Nano Novin Polymer Company (Sari, Iran). Ferric (III) chloride hexahydrate 96% and ferrous (II) chloride 98% were purchased from Sigma-Aldrich (Castle Hill, Australia), as well as acrylic acid, starch, ammonium persulfate, and sodium hydroxide (NaOH). All other chemicals obtained were of analytical grade and were used in their original form unless otherwise noted.

### 2.2. In Situ Precipitation of Fe_3_O_4_ Nanoparticles on Nanochitin

Fe_3_O_4_ nanoparticles were synthesised on the surface of nanochitin using the co-precipitation of ferric (III) chloride hexahydrate and ferrous (II) chloride as the iron source. We used 500 mL of distilled water to dilute a suspension of nanochitin. To prevent oxidation, the mixture was stirred for a further hour while being continuously purged with nitrogen. Finally, 2.2 g of ferric (III) chloride hexahydrate was added. After a further hour of stirring, 0.45 g of ferrous (II) chloride was added to the dispersion. After gradually adding 1 M NaOH solution, Fe_3_O_4_ nanoparticles were successfully synthesised on nanochitin. Once the mixture becomes black, the reaction is complete. The synthesised magnetic nanochitin was prepared by performing three 15-min centrifugation cycles in distilled water to remove unreacted compounds, followed by homogeneously re-dispersing the solution in distilled water using ultrasonic treatment. Finally, the resulting suspension was capped and cryopreserved at 4 °C.

### 2.3. Synthesis of Starch-g-Acrylic Acid/Magnetic Nanochitin

Inspired by the method developed by Ebru et al. [30], we then fabricated our ferrogels. To do so and under a nitrogen environment, gelatinised starch containing different concentrations of magnetic nanochitin (0.1, 0.5, 1, 1.5, and 2 wt.%) was grafted with acrylic acid in a 500 mL round bottom flask. Gelatinised starch was prepared by heating 4 g of starch and 100 mL of distilled water to 90 °C while stirring continually for 1 h. To facilitate free radical formation on starch, the gelatinised starch was cooled to 35 °C and treated with 50 mg APS for 15 min. After this, we added acrylic acid at a monomer/starch weight ratio of 1.5 to the mixture of gelatinised starch/magnetic nanochitin to fabricate the ferrogels containing different concentrations of magnetic nanochitin (0.1, 0.5, 1, 1.5, and 2 wt.%). The resulting ferrogels were rinsed with an ethanol–water solution (50:50 *v*/*v*) to eliminate the unreacted monomers after ferrogelation at room temperature.

### 2.4. Characterisation of Starch-g-Acrylic Acid/Magnetic Nanochitin Ferrogels 

The nanostructured morphologies of nanochitin and magnetic nanochitin were analysed using scanning electron microscopy (SEM, Zeiss) and transmission electron microscopy (TEM) on a JEOL 2100. Each sample was first diluted with ethanol to a concentration of about 0.0015 wt.%, and then the suspension was sonicated to confirm uniformity. Once the excess moisture was removed, the samples were cast onto carbon-coated perforated grids for drying. Image analysis software called ImageJ was used to examine the micrographs. The magnetic properties of magnetic nanochitin were measured using a Quantum Design MPMS-5 T SQUID magnetometer. The temperature was set to 300 K, and the range of the magnetic field was varied between −20,000 and 20,000 Oe. To investigate the microstructured morphologies of the loaded magnetic nanochitin in the freeze-dried ferrogels, we utilised SEM, Zeiss, Supra, Oberkochen, Germany. In order to perform FTIR analysis on nanochitin, magnetic nanochitin, and ferrogel, a Bruker Vertex 70 FTIR was used. A universal tensile testing machine (Instron; Norwood, MA, USA) with a 200 N load cell was used to analyse the tensile stress and mechanical characteristics. After applying a silicone coating, samples measuring 10 mm in width, 6 mm in thickness, and 35 mm in length were subjected to mechanical testing at a continuous stretching rate of 60 mm min^−1^. We measured the elongation at break to study the deformation and fracture strain of ferrogels. The area under the stress-strain curves was used to determine the toughness or the hydrogel’s capacity to absorb energy before cracking. The self-healing characteristics of the ferrogels were visually observed by slicing them and then touching them from the cutline. The healing efficiency was determined based on the stress ratios of healed and original ferrogels. Using a current monitoring device with a light-emitting diode (LED) bulb as an indicator, the self-repair and responsiveness of ferrogel’s electrical properties to changes in resistance were shown in real-time. The resistance in the ferrogel was measured using a multimeter. To test how well the magnetic sensing was working in real-time, we placed the ferrogel between two probes and monitored its electric resistance over time while exposed to a permanent external magnet. To mimic human skin, the ferrogel pen was used as a touch-screen stylus.

## 3. Results and Discussion

### 3.1. Nanostructure, Microstructure, Chemical Structure, and Ferrogelation 

**Figure 1:**
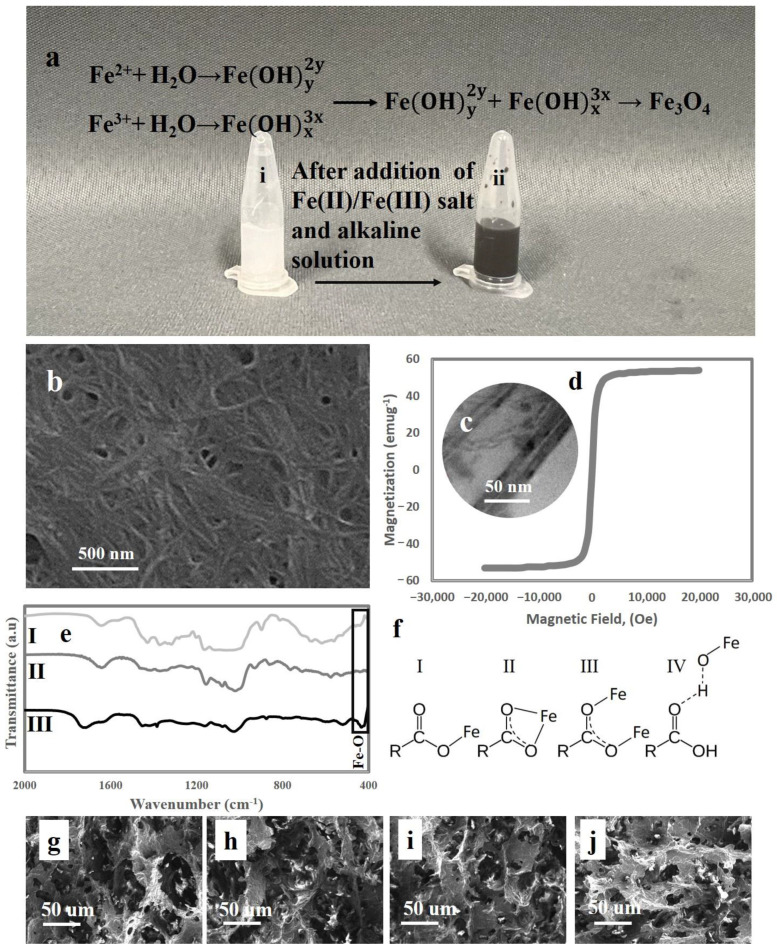
(**a**) Synthesis of magnetic nanochitin at a stoichiometric aqueous quantity of Fe(II)/Fe(III) salt solution and an alkaline solution in a non-oxidizing environment; (**b**) SEM photograph of nanochitin; (**c**) TEM photograph of magnetic nanochitin; (**d**) magnetic hysteresis loop of magnetic nanochitin at the temperature of 300 K; (**e**) FTIR spectra of (I) magnetic nanochitin, (II) nanochitin, and (III) acrylic acid-g-starch/magnetic nanochitin ferrogel; (**f**) the possibility for interaction between iron oxide nanoparticles and polymeric chains; (**g**) SEM photographs of ferrogel containing 0.5 wt.% magnetic nanochitin; (**h**) SEM photographs of ferrogel containing 1 wt.% magnetic nanochitin; (**i**) SEM photographs of ferrogel containing 1.5 wt.% magnetic nanochitin; and (**j**) SEM photographs of ferrogel containing 2 wt.% magnetic nanochitin.

Using a stoichiometric aqueous quantity of Fe(II)/Fe(III) salt solution and an alkaline solution in a non-oxidizing environment, magnetic nanochitin was synthesised, as shown in Figure 1a. It has been observed that the magnetic characteristics of Fe_3_O_4_ nanoparticles lie between 30 and 85 emug^−1^, but it tends to aggregate due to van der Waals forces among the nanoparticles to decrease their surface energy [31]. This is the key difficulty in synthesising Fe_3_O_4_ nanoparticles using the co-precipitation technique. In this research, we used nanochitin as a template onto which we could graft nanoparticles of Fe_3_O_4_ to minimise this behaviour. Nanochitin fibres’ abundance of surface functional groups helps keep Fe_3_O_4_ nanoparticles dispersed in the suspension. When the suspension’s colour changed from yellow to black, we reckoned the reaction had taken place. More importantly, after a month in an aqueous medium, the Fe_3_O_4_ nanoparticles that had been grafted onto nanochitin remained in stable suspension. When tested with a permanent magnet, magnetic nanochitin was shown to have a positive reaction, suggesting that it displays a magnetic response (Video S1). The images using TEM demonstrate that the diameter of nanochitin is on the nanoscale, with an average diameter of 53 nm (Figure 1b). As demonstrated in the TEM image (Figure 1c), the functional groups on nanochitin can serve as nucleation sites for Fe_3_O_4_ nanoparticle deposition, confirming that Fe_3_O_4_, instead of aggregation, had efficiently grafted onto the surface of nanochitin. Magnetic nanochitin also exhibited typical superparamagnetic behaviour with a typical S-shaped hysteresis loop, and its magnetisation rises with increasing external magnetic strength, as shown in Figure 1d, which also reveals a magnetic saturation value of 51.47 emu/g^−1^. This is another confirmation demonstrating that the Fe_3_O_4_ nanoparticles were successfully grafted onto the nanochitin surface.

FTIR spectroscopy was used to examine the magnetic properties created by grafting Fe_3_O_4_ nanoparticles onto nanochitin (Figure 1d). The FTIR spectra of magnetic nanochitin and nanochitin (Figure 1d, I and II) show multiple similar peaks. The main distinguishing feature of chitin structures is the formation of intermolecular hydrogen bonds, which can be attributed to the peak at 1658 cm^−1^ as the formation of intermolecular hydrogen bonds CO…HN. The peak at 1179 cm^−1^ can be assigned to amide I, which is caused by the flexural bending of the NH bonds, whereas the peak at 1560 cm^−1^ can be assigned to amide II. Additionally, the C-O-O bond vibration inside the chitin rings was linked to the 1075 cm^−1^ peak. The typical bandwidth of magnetic nanochitin is about 507 cm^−1^, as seen in Figure 1d, I. This sheds light on the existence of Fe_3_O_4_ nanoparticles on the surface of nanochitin and the successful interactions between the functional groups of nanochitin and Fe_3_O_4_ nanoparticles [32]. Finally, we fabricated the ferrogels by graft radical copolymerizing starch and acyclic acid monomers in the presence of previously synthesised magnetic nanochitin in situ. As just said, the 507 cm^−1^ absorption peak is identical to the Fe-O bond in magnetic nanochitin, and the adsorption of negatively charged polyelectrolytes onto iron oxide nanoparticles slightly shifts this band to a new high at 514 cm^−1^. The presence of the intense bands at 1713 ^1^ and 1743 cm^−1^ in the spectra of magnetic nanochitin-loaded starch-g-acyclic acid may be attributed to the presence of carbonyl groups, revealing the successful interaction of magnetic nanochitin with the polymer chains. It also functioned as a crosslinking agent in the construction of a 3D network structure that could trap water molecules through interactions between polymeric chains and Fe_3_O_4_ nanoparticles. Hence, it may replace N,N′-methylene bisacrylamide, which is an irreversible static chemical cross-linker during the fabrication of starch-g-acyclic acid ferrogel. A permanent magnet was used to mimic the existence of a magnetic response in the ferrogel, and it was revealed that the ferrogel, due to the presence of Fe_3_O_4_ nanoparticles, can swim in water when directed by magnetism. As a result, magnetic nanochitin gave our ferrogels magnetic characteristics that can attract a typical permanent magnet (Video S2). The negatively charged carboxyl groups in poly(acyclic acid) have been theorised in the literature to be adsorbed onto the exposed Fe atoms on the surface of Fe_3_O_4_ nanoparticles grafted on nanochitin, establishing stable ionic bonds [33,34]. Figure 1e depicts the possible coordination of a carboxyl group with an iron atom (I, II, and III). The positively charged H^+^ ions may also be absorbed by the oxygen atoms on the surface of the Fe_3_O_4_ nanoparticles. If the remaining carboxylic/carboxylate groups in starch-g-acyclic acid/magnetic nanochitin interact with OH/OH^2+^ as the attractive force, structure IV in Figure 1e is conceivable. As a result, we hypothesise that the physical crosslinking and ferrogelation of acyclic acid-g-starch/magnetic nanochitin are caused by ionic or dipole interactions at ambient temperature and neutral pH [34,35]. By varying the concentration of magnetic nanochitin, the porous structure of ferrogels is exhibited in Figure 1f–i. The magnetic nanochitin concentration was shown to be related to narrower pore diameters with an average pore size of 39 μm in ferrogel containing 1 wt.% magnetic nanochitin and 17 μm in ferrogels containing 2 wt.% magnetic nanochitin, most likely due to enhanced crosslinking in the ferrogels.

### 3.2. Mechanical Properties, Self-Healing, and Notch Insensitivity

**Figure 2:**
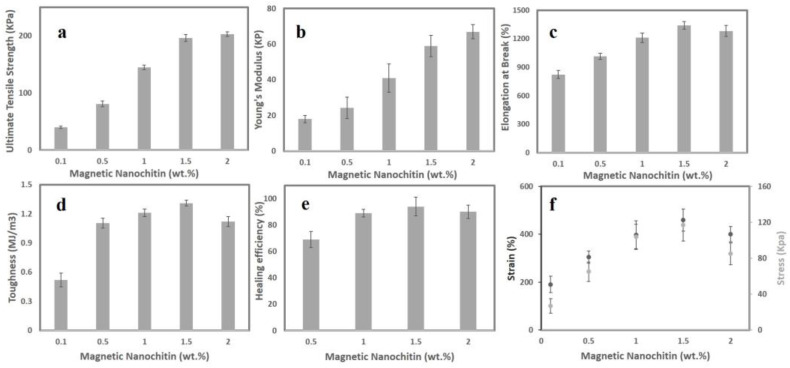
(**a**) Ultimate tensile strength for ferrogels at different magnetic nanochitin concentrations; (**b**) Young’s modulus for ferrogels at different magnetic nanochitin concentrations; (**c**) elongation at break of ferrogels at different magnetic nanochitin concentrations; (**d**) toughness of ferrogels at different magnetic nanochitin concentrations; (**e**) tensile strength healing efficiency of ferrogels at different magnetic nanochitin concentrations; and (**f**) notch sensitivity analysis of ferrogels at varying concentrations.

During the ferrogelation, we demonstrated that magnetic nanochitin might be used as a cross-linker alternative to N,N′-methylene bisacrylamide. In addition, it has been suggested that adding nanofillers to the starch network may boost the material’s tensile strength. Given that starch is naturally a brittle polymer, graft copolymerisation of vinyl monomers, such as acrylic acid, can be a straightforward and efficient way to increase the mechanical strength of starch. Here, we conducted tensile stress experiments on rectangular shape samples to demonstrate the effect of magnetic nanochitin on the mechanical characteristics of starch-g-acrylic acid ferrogels. The results of mechanical tests for the ferrogels containing 0.1, 0.5, 1, 1.5, and 2 wt.% of magnetic nanochitin are shown in Figure 2. As seen, by increasing the concentration of magnetic nanochitin, the ultimate tensile strength (Figure 2a) and Young’s modulus (Figure 2b) has steadily increased, indicating that magnetic nanochitin has a proper impact on reinforcing the mechanical properties of the ferrogels. The elongations at break of the ferrogels have also increased, leading to a stretchability of 1341% at 1.5 wt.% magnetic nanochitin. With more than 1.5 wt.% magnetic nanochitin, however, the elongations at break decreased slightly, most likely due to the presence of some agglomerations inside the ferrogel’s network, which functions as stress concentration points and reduces the ferrogels’ stretchability under strain. Measuring toughness using the mechanical test was performed on all ferrogels at a variety of magnetic nanochitin concentrations (0.1, 0.5, 1, 1.5, and 2 wt.%) to determine which hydrogel had the highest toughness since this is one of the most important criteria for a wearable strain sensor. The toughness of the ferrogel reinforced with 1.5 wt.% magnetic nanochitin is 1.31 MJ/m^3^. However, the toughness decreases with increasing concentrations of magnetic nanochitin, which may be owing to an increased degree of crosslinking (Figure 2d).

We hypothesised that polymer chains would be able to entangle together over the surface of a newly cut ferrogel and form inter-chain bonds with Fe atoms since no chemical static cross-linker was used in the manufacturing of our suggested ferrogels. Here, we postulate that the carboxyl groups would adsorb onto the exposed Fe atoms of the surface of Fe_3_O_4_ via three discussed dynamic potential coordination bonds (I, II, and III), or that the surface oxygen atoms of the Fe_3_O_4_ would be able to take in H^+^ ions, where the surface of OH/OH^2+^ species can participate with the remaining carboxylic/carboxylate groups in the ferrogel to form structure IV in Figure 1e. It eventually leads to the dynamic behaviour of ferrogels by which the damaged section of the ferrogel can be healed and bonded together again at room temperature without the need for any external stimulation, additives, or force. Ferrogels with varying amounts of magnetic nanochitin self-healed completely and coalesced into a single structure. Figure 2e shows the optimum mechanical strength efficiency of 93% for the ferrogel containing 1.5 wt.% magnetic nanochitin. This illustrates that after 6 h of self-healing, the new connections at the interface are robust enough to span the space between two sliced ferrogels. Furthermore, as shown in Video S3, after 6 h of self-healing of the ferrogel containing 1.5 wt.% magnetic nanochitin with multiple damages and cuts, this ferrogel is incredibly twistable and stretchy. These results support the hypothesis that ferrogels may self-heal while maintaining great performance. 

Next, notches were applied to each of the ferrogels in order to evaluate the notch sensitivity of the starch-g-acrylic acid/magnetic nanochitin ferrogels at varying concentrations. This was performed before the ferrogels were stretched under tensile stress. According to the findings, the ferrogel that had been fabricated with 1.5 wt.% magnetic nanochitin had the highest level of notch insensitivity and maintained an impressive level of stability and blunting throughout the whole experiment. One of the reasons why ferrogels’ toughness behaviour may make them resistant to cracking is because of the presence of dynamic, reversible bonds in the ferrogels. It was determined that the ferrogel containing 1.5 wt.% magnetic nanochitin would be the best sample with which to continue the research based on the outcomes of the mechanical, notch insensitivity, and self-healing tests. These tests determined that the material was not sensitive to notches and may have the needed resilience for strain-sensing applications without the need for additional irreversible static chemical crosslinks.

### 3.3. Conductivity and Strain Sensing

**Figure 3:**
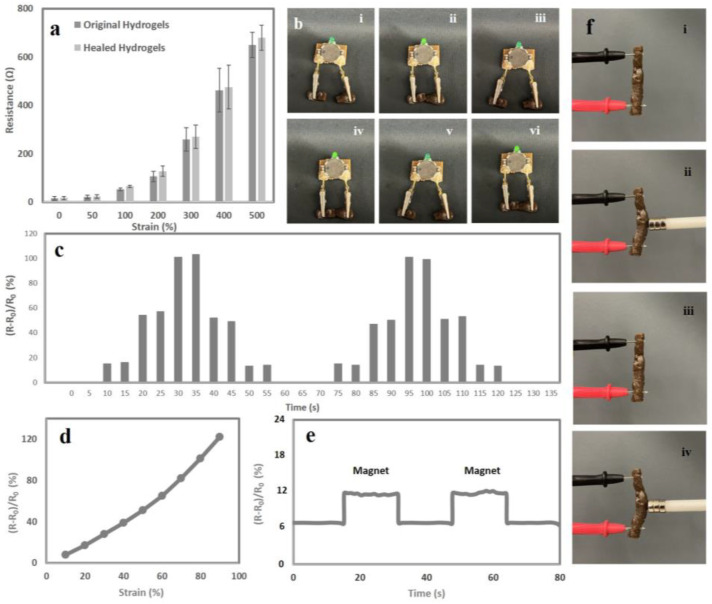
(**a**) Resistance response to different strains for original and healed ferrogels; (**b**) electrical recovery of ferrogel after (i) disconnection, (ii) connection, (iii) disconnection, (iv) connection, (v) disconnection, and (vi) connection from the cutline; (**c**) the real-time resistance change detection of a ferrogel connected to an artificial wooden elbow at various angles; (**d**) gauge factor curve of the ferrogel strain sensor containing 1.5 wt.% magnetic nanochitin; (**e**) using the ferrogel as a magnetic strain sensor by exposing the ferrogel to the external magnet; (**f**) photographs of magnetic sensitivity of the ferrogel (i and iii) before exposing to a permanent magnet and (ii and iv) after exposing to a permanent magnet.

If a soft, notch-insensitive, tough, and stretchy ferrogel can maintain conductivity, then it might be an excellent candidate for being employed in flexible electronics. Given the large number of ionic connections that are present in ferrogels, it was reasonable to assume that our ferrogels would exhibit ionic conductivity. Ferrogels with varied concentrations of magnetic nanochitin were subjected to a current monitoring system that was equipped with an LED light indicator. This system displayed the ionic conductivity, strain sensitivity, and electrical self-healing capabilities of the ferrogels in real time. When the ferrogels were affixed to the device, the LED light began to illuminate. It was observed that applying stress to the ferrogel and stretching it caused the LED’s brightness to drop; however, once the tension was released, the brightness of the LED reverted to normal. It is possible that the occurrence might be explained by the fact that the stretching process induces a distortion in the network structure of the ferrogel (Video S4). The ferrogel was then deformed and stretched, which caused the contact area between the ionic bonds that make the material conductive to decrease. This caused the material to become less conductive. As a consequence of this, the resistances of the original ferrogel and the stretched ferrogel were distinct, as shown by the ratio (R − R_0_)/R_0_ in Figure 3a. Due to the self-healing and rapid reconstruction of the 3D conductive network of the ferrogels, the electrical current in the ferrogels was able to quickly recover after being cut in half, as was demonstrated by the LED light in Figure 3b. In particular, the cut ferrogel restored its ionic conductivity immediately due to the instantaneous self-healing of the cut area, which suggests that ferrogels are well suited for applications such as emergency circuit repair, electrical circuit building, switching functions, and electric lines (Figure 3b).

The ferrogel also showed its efficacy as a bendable and soft strain sensor by connecting to an artificial wooden elbow at various angles and strain sensing in real-time. As the strain and bending angles were changed, the strain sensor’s resistance increased to varying degrees. Figure 3c reveals that the internal 3D network of the ferrogels can quickly repair itself from damage caused by the stretching, and the strain sensor returned to its initial value by revealing the same values for the relative resistance (R − R_0_)/R_0_ after the elbow were straightened, proving a consistent, measurable electrical stability for detecting resistance in real-time at different bending degrees. If the strain was maintained at the same level for an extended period of time, the resistance would stay at the same level (Figure 3c). One of the most crucial factors in establishing the sensitivity of a strain sensor is the gauge factor, often known as GF. This may also accurately reflect the sensing capability of the sensor. The GF for this ferrogel was calculated by plugging the available data into the formula GF = (R/R_0_)/ε. Strain is represented by ε, while R_0_ and R indicate the resistance at zero strain and the relative resistance variation with stretch, respectively. Figure 3d displays the GF values of 0.5 and 1.40 for a 10–120% tensile strain. The GF of the ferrogel has been shown to be within the range that is considered optimal for strain-sensing applications (Table 1). Therefore, we hypothesise that the ferrogel might satisfy the needs of a strain sensor.

Because the ferrogel has magnetic properties, it is possible to use it as a magnetic sensor. In order to evaluate the ferrogel’s capability of detecting magnetic strain, it was placed between the probes of a multimeter and then exposed to an external magnet (Figure 3e). As seen, the ferrogel was able to quickly sense strain and respond to external actuation by moving and bending as a result of this property. When an external magnetic field was applied to the ferrogel, the changing resistance, as well as the real-time resistance track, was revealed. If the strain sensor returns back to where it was before the external magnet was removed, this indicates that the electrical stability that is necessary for sensing resistance in real-time has been restored (Figure 3f). Ferrogel has the potential to imitate the look, feel, and function of human skin when it is employed as an electronic skin. Ferrogel’s bright future as a prosthetic finger in flexible electronics, electronic skin for robotics, and human–machine interfaces is shown by utilising it as a touch-screen pen to draw PEJMAN letters on an iPad. This demonstrates Ferrogel’s potential applications in these areas (Video S5).

## 4. Conclusions

The performance of hydrogels is directly influenced by their crosslinking structures. The creation of such hydrogels is made more difficult as a result of the fact that a mix of crosslinks is often necessary to produce the three-dimensional network of hydrogels with both the autonomous self-healing and the durability required for applications involving strain sensing. In contrast, this research makes use of a straightforward method to produce a stretchy, magneto-responsive, ionic conductive ferrogel for strain sensing. This is accomplished without the use of any crosslinking agents. The ferrogel showed the ability to self-heal when exposed to air and displayed strain sensing in real time when it was activated by an external magnetic field. Ferrogel proved to be an effective touch-screen pen, as well, according to our findings. Our discoveries might pave the way for more effective techniques for developing multifunctional hydrogels that can be used for a broad variety of applications such as flexible strain sensors and health monitors, as well as soft robotics.

## Figures and Tables

**Table 1:** Comparison of the present hydrogel’s sensing sensitivity to previously reported sensing sensitivity for different hydrogels.

Polymers	Strain Sensing Range (%)	Gauge Factor	Ref.
Sodium alginate/tannic acid/polyacrylamide hydrogels	0.05–100	2	[36]
Polyaniline/poly(4-styrenesulfonate)-20UPy hybrid hydrogels	~300	3.4	[37]
F-Poly(*N*-isopropyl acrylamide)/polyaniline hydrogels	0–120	3.9	[6]
Polyacrylamide/carboxymethyl cellulose/Fe^3+^ hydrogel	0–100	1.4–2.6	[38]
Multi-walled carbon nanotubes/polyacrylamide hydrogels	50–200	4.02–5.67	[39]
Polypyrrole/polyacrylamide hydrogels	~200	1.25	[7]
Polyvinyl alcohol-tannic acid-eutectic gallium-indium hydrogels	0–50	2.59	[8]
Lignin reinforced hydrogels	0–51.5	2.51	[40]
CMC-l-OCNF-l-ChNF-l-TA:Fe^III^ hydrogel	0–55	2.69	[11]
Magnetic nanochitin ferrogel	10–120	0.5–1.4	This work

## Data Availability

Not applicable.

## Supplementary Materials

The following supporting information can be downloaded at: https://www.mdpi.com/article/10.3390/s23031138/s1, Video S1: Using a permanent magnet to show the magnetic nanochitin displays a magnetic response; Video S2: Using a permanent magnet to mimic the existence of a magnetic response in the ferrogel; Video S3: Twisting and stretching a 6 h self-healed ferrogel containing magnetic nanochitin with multiple damages and cuts; Video S4: Stretching the ferrogel affixed to the device with an the LED light; Video S5: Utilising the ferrogel as a touch-screen pen to draw PEJMAN letters on an iPad.
